# β-Catenin Is a Positive Regulator of Estrogen Receptor-α Function in Breast Cancer Cells

**DOI:** 10.3390/cancers3032990

**Published:** 2011-07-22

**Authors:** Nibedita Gupta, Fee Schmitt, Sina Grebhardt, Doris Mayer

**Affiliations:** Hormones and Signal Transduction Group, German Cancer Research Center (DKFZ), DKFZ-ZMBH Alliance, Im Neuenheimer Feld 581, 69120 Heidelberg, Germany; E-Mails: n.gupta@dkfz.de (N.G.); fee.schmitt@gmx.de (F.S.); s.grebhardt@dkfz.de (S.G.)

**Keywords:** β-catenin, estrogen receptor, cross-talk, gene expression, transcriptional activity, breast cancer cells

## Abstract

Estrogen receptor-alpha (ERα) is a key factor in the development of breast cancer in humans. The expression and activity of ERα is regulated by a multitude of intracellular and extracellular signals. Here we show a cross-talk between β-catenin and ERα in human breast cancer cells. Knockdown of β-catenin by RNAi resulted in significant reduction of ERα mRNA and/or protein levels in MCF-7, T-47D, and BT-474 breast cancer cells and in significant reduction of estradiol-induced expression of the ERα target genes pS2 and GREB1. In addition β-catenin silencing resulted in significant decrease of growth of MCF-7 cells both in the absence and presence of estradiol. β-catenin and ERα could not be co-immunoprecipitated by ERα antibodies from lysates of E2-treated or untreated cells suggesting lack of direct physical interaction. It is concluded that β-catenin is a positive regulator of ERα mRNA and protein expression.

## 1. Introduction

Estrogen receptor-α (ERα) is a key regulator of proliferation, growth, differentiation, development and maintenance of a wide range of tissues including the mammary glands. Furthermore, it has been implicated in various pathological processes including breast cancer. The receptor is mainly activated by 17β-estradiol (E2) and functions as a transcription factor to regulate a wide range of cellular activities in response to E2. The regulation of ERα transcriptional activity is highly complex and not yet fully understood. Ligand-activated ERα dimerizes and typically binds to specific estrogen responsive elements (ERE) in the promoter regions of target genes, although ERE-independent mechanisms of ERα action have also been reported [1–3]. Furthermore, considerable evidence has been accumulated during the last decade showing that ERα can be activated by E2-independent mechanisms. A number of growth factor activated signaling pathways and their downstream effector protein kinases have been reported to modulate ERα activity by phosphorylation at specific serine residues [4–7].

We have previously shown that in estrogen-responsive MCF-7 breast cancer cells, glycogen synthase kinase-3β (GSK-3β), a serine kinase involved in the regulation of a multitude of cellular functions, interacts with and stabilizes ERα in the cytoplasm of cells in the absence of E2 [8,9]. Treatment of the cells with E2 results in rapid activation of Akt/PKB and - as a consequence - in phosphorylation and inactivation of GSK-3β. Inactivation of GSK-3β results in release of ERα and translocation of the receptor into the nucleus. Interestingly, Cardona-Gomez *et al.* [10] reported an interaction and complex formation of ERα, GSK-3 and β-catenin in the hippocampus of the adult female rat and a release of β-catenin from this complex in the presence of the hormone. Kouzmenko [11] observed co-immunoprecipitation of ERα and β-catenin from HCT116 human colon cancer cells which had been transfected with FLAG-ERα, both in the absence and presence of E2.

The first report on functional interaction between β-catenin and a nuclear receptor was published by Truica *et al.* [12] who identified β-catenin as co-activator of the androgen receptor. Later on, other nuclear receptors including ERα were reported to interact with the Wnt/β-catenin/Tcf signaling pathway [13]. In addition, β-catenin was frequently found dysregulated in breast cancer [14]. However, the potential cross-talk mechanisms between β-catenin and ERα have not yet been studied in detail in breast cancer. Therefore, we were interested to investigate the potential physical and functional interaction between β-catenin and ERα in breast cancer cells.

## 2. Results and Discussion

### 2.1. β-Catenin Translocates to the Nucleus upon E2 Treatment but does not Physically Interact with ERα

ERα has been known for a long time to be localized both in the nucleus and cytoplasm in unstimulated ERα-positive breast cancer cells and treatment with E2 results in rapid nuclear translocation of cytoplasmic ERα. In the present study, we first investigated whether E2 treatment has an impact on intracellular β-catenin localization and whether there is a physical interaction of β-catenin and ERα in breast cancer cells.

**Figure 1.**
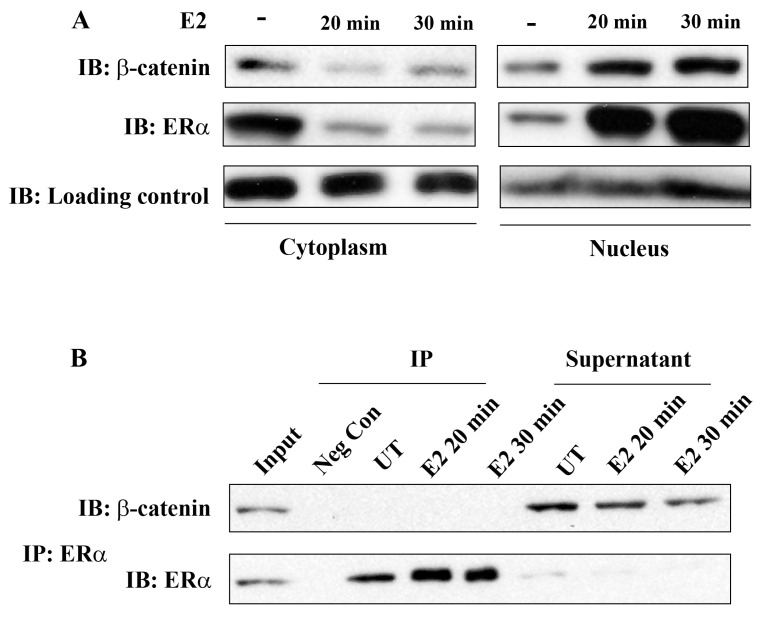
E2 treatment causes nuclear translocation of β-catenin in MCF-7 cells. (A) MCF-7 cells remained untreated or were treated for 20 and 30 min with 10 nM E2. Thereafter, the cells were fractionated and cytoplasmic and nuclear fractions were analyzed for β-catenin and ERα by immunoblotting (IB). eFI4B was used as loading control for the cytoplasmic fraction and Histone H3 for the nuclear fraction; (B) MCF-7 cells remained untreated (UT) or were treated for 20 and 30 min with 10 nM E2. Thereafter ERα was immunoprecipitated (IP) from cell lysates. The immunoprecipitate (left side) contains ERα but β-catenin was not co-precipitated. Supernatant after IP (right side) contains β-catenin but only traces of ERα. Neg Con, negative control containing non-immune IgG.

Cell fractionation studies clearly showed the presence of ERα both in the cytoplasm and nucleus in MCF-7 cells that were not stimulated with E2 and almost complete ERα translocation into the nucleus occurred within 20 min of E2 treatment. Interestingly, β-catenin also translocated into the nucleus under these experimental conditions (Figure 1A). This observation suggests the potential role of β-catenin in E2/ERα signaling. However, ERα and β-catenin did not co-immunoprecipitate, neither in unstimulated nor in E2 stimulated cells. Figure 1B shows almost complete immunoprecipitation of ERα by anti-ERα antibodies, but β-catenin remained in the supernatant under these conditions. Similar results were obtained with T-47D cells (Suppl. Figure 1). We conclude that ERα and β-catenin do not physically interact in the breast cancer cells studied. The mechanisms related to E2 induced nuclear translocation of β-catenin and the potential role of GSK-3 in this process are not known and will not be further addressed in this study.

### 2.2. β-Catenin Knockdown Results in Reduced ERα mRNA and Protein Levels

**Figure 2.**
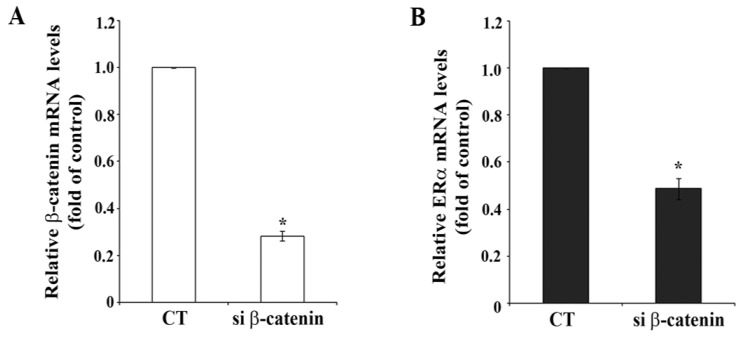
β-catenin knockdown results in reduction of ERα mRNA levels in MCF-7 cells. MCF-7 cells were transfected with CT siRNA (CT) or β-catenin siRNA (si β-catenin). β-catenin (A) and ERα (B) mRNA levels were quantified by quantitative RT-PCR. HMBS was used as internal control. Data represent the mean ± SEM of the results from three independent experiments (* P < 0.05).

In order to get more insight into the potential functional interaction between β-catenin and ERα activity β-catenin was down-regulated by transfection of siRNA specifically targeting β-catenin. Figure 2A shows a 70% reduction of β-catenin mRNA level in MCF-7 cells after β-catenin siRNA transfection compared to cells transfected with non-targeting siRNA (CT siRNA). Importantly, ERα mRNA level was significantly reduced by about 50% under the same conditions (Figure 2B).

**Figure 3.**
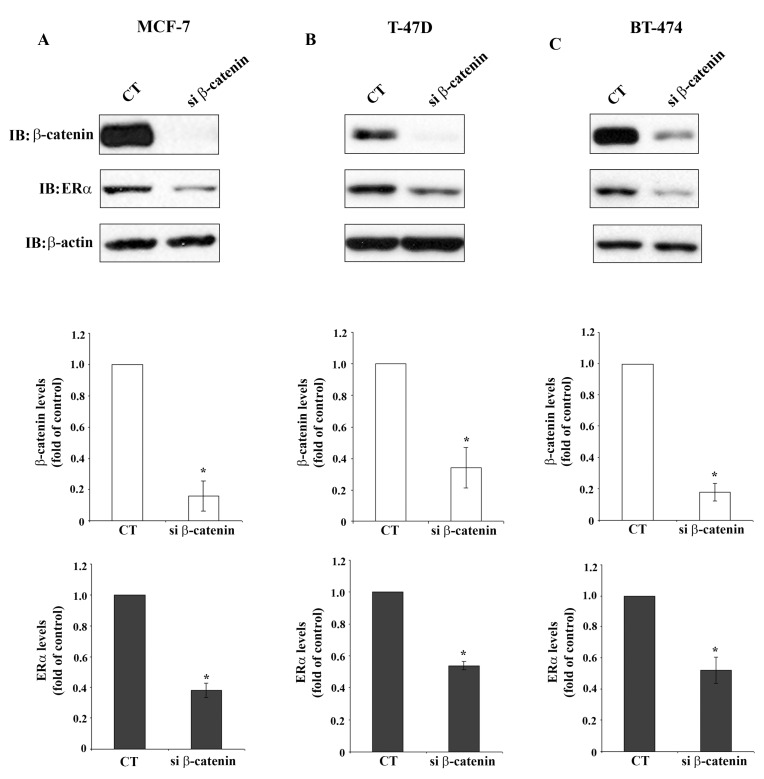
β-catenin knockdown results in reduction of ERα protein levels in MCF-7 (A), T-47D (B) and BT-474 (C) cells. Cells were transfected with CT siRNA or β-catenin siRNA. Cell lysates were analyzed by immunoblotting for β-catenin and ERα protein levels. β-actin was used as loading control. Figures in the lower panels each show quantitative analyses of β-catenin (middle row) and ERα (bottom row) immunoblots. Data represent the mean ± SEM of three independent experiments (* P < 0.05).

Figure 3 demonstrates significant reduction of β-catenin protein levels in MCF-7 cells, T-47D cells and BT-474 cells after β-catenin siRNA transfection, determined by Western blot analysis. Reduction of β-catenin protein levels is accompanied by significant reduction of ERα protein levels in the three cell lines, which agrees with the reduction of the respective mRNA shown in Figure 2. This observation indicates regulation of ERα expression by β-catenin which may occur on the transcriptional level or by stabilization of ERα mRNA. This is a novel finding suggesting a so far unknown cross-talk between β-catenin and ERα signaling pathways in breast cancer cells.

**Figure 4.**
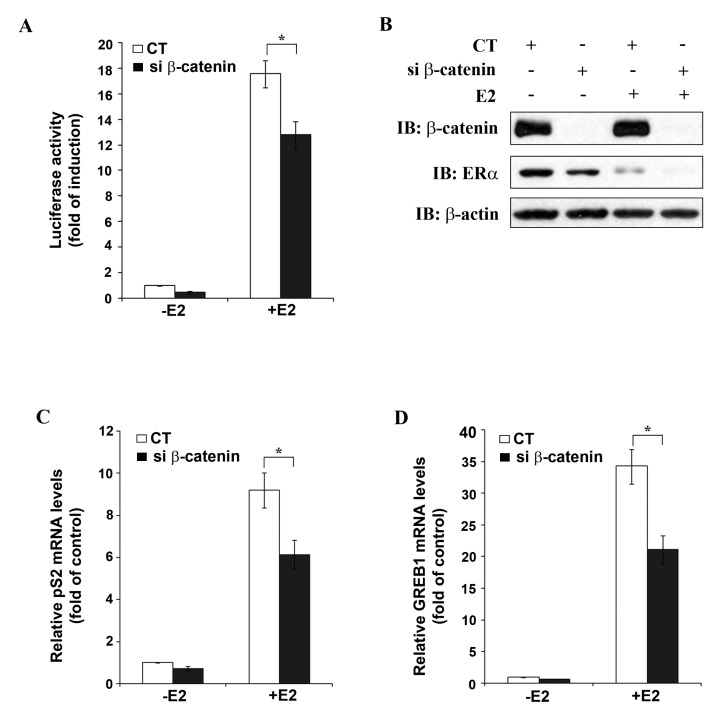
β-catenin knockdown results in reduction of ERα transcriptional activity. (**A**, **B**) MELN cells were transfected with CT siRNA or β-catenin siRNA and then remained untreated or were treated with 10 nM E2 for 16 h. (**A**) Luciferase activity; (**B**) Western blot analysis of lysates from (**A**); (**C**, **D**) MCF-7 cells were transfected with CT siRNA or β-catenin siRNA and then remained untreated or were treated with 10 nM E2 for 24 h. (**C**) and (**D**) show pS2 and GREB1 mRNA levels quantified by qRT-PCR. Data represent the mean ± SEM of the results from three independent experiments (* P < 0.05).

Interestingly, knockdown of ERα by transfection of siRNA specifically targeting ERα did not affect β-catenin mRNA or protein levels (data not shown). Furthermore, E2 treatment did not have an influence on β-catenin levels (see Figure 4B). It may be concluded that β-catenin is a regulator of ERα expression, but ERα is not a regulator of β-catenin in MCF-7 cells.

### 2.3. β-Catenin Down-Regulation Results in Reduced ERα Transcriptional Activity

To further study β-catenin as a potential positive regulator of ERα function, MELN cells (derived from MCF-7 cells by stable transfection with an ERE-controlled luciferase reporter plasmid) were transfected with either CT siRNA or β-catenin siRNA, followed by stimulation with 10 nM E2 and performance of luciferase assay. In CT siRNA transfected cells, about 18-fold increase in luciferase activity was observed after E2 treatment (Figure 4A), whereas after transfection of β-catenin siRNA, E2 treatment resulted in only 13-fold increase in luciferase activity, which represents 27% inhibition of E2-induced luciferase activity by β-catenin silencing under the experimental conditions used.

Figure 4B shows Western blot analysis of the MELN cell lysates used for luciferase assay. β-catenin siRNA transfection resulted in complete knockdown of β-catenin (lanes 2 and 4) and in partial down-regulation of ERα, as observed before (Figure 3). In agreement with earlier reports [15,16] E2 treatment resulted in down-regulation of ERα mRNA expression in CT siRNA transfected cells (lane 3). The ability to undergo ligand-induced down-regulation is a characteristic of ERα and represents a physiologically important feedback mechanism to limit hormone action in target tissues. β-catenin siRNA transfection in presence of E2 enhanced down-regulation of ERα (lane 4). As already mentioned E2-treatment did not affect β-catenin protein levels (Figure 4B). The role of β-catenin in the regulation of ERα transcriptional activity was confirmed by studying the effect of β-catenin knockdown on the expression of two well-established endogenous ERα target genes, pS2 [17] and GREB1 [18]. Quantitative real-time PCR (qRT-PCR) analysis revealed a significant decrease in E2-induced expression of both pS2 (Figure 4C) and GREB1 (Figure 4D) after β-catenin silencing in MCF-7 cells. These results reassert β-catenin as a positive regulator of ERα target gene expression.

### 2.4. β-Catenin Down-Regulation Results in Reduced Cell Growth

**Figure 5.**
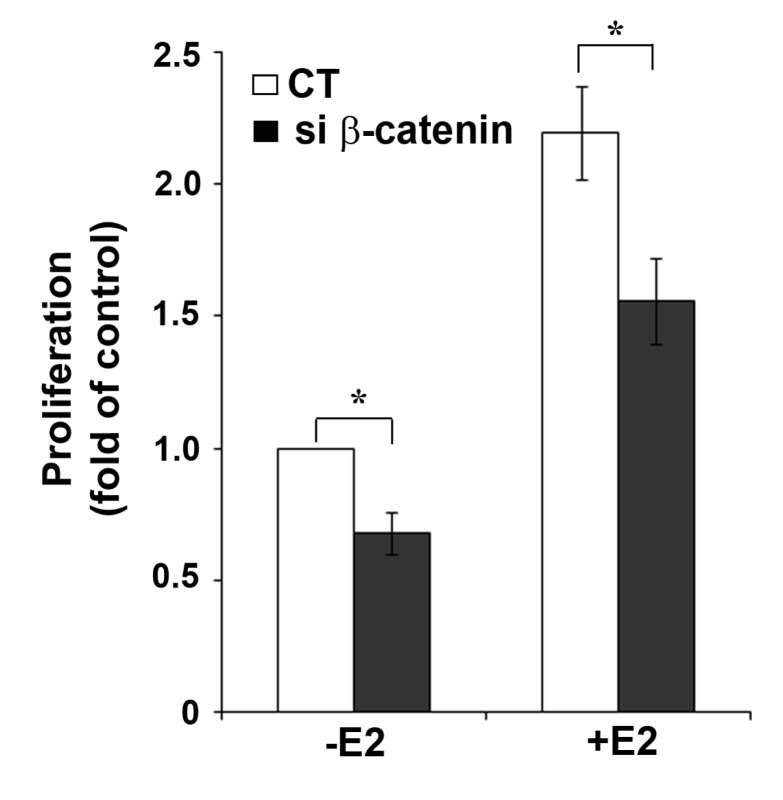
β-catenin knockdown reduces E2-induced growth of MCF-7 cells. MCF-7 cells were transfected with non-targeting siRNA (CT) or β-catenin siRNA and then remained untreated or were treated with 10 nM E2 for 72 h. Cell proliferation was analyzed using the crystal violet assay. Data represent the mean ± SEM of three independent experiments (* P < 0.0 5).

The functional interaction between β-catenin and ERα activity was further confirmed by showing the inhibitory effect of β-catenin down-regulation on growth of MCF-7 cells (Figure 5). Proliferation of MCF-7 cells can be stimulated by growth factors [19], but depends largely on ERα activity. Interestingly, in both untreated and E2-treated MCF-7 cells, transfection of β-catenin siRNA resulted in similar reduction of cell growth (32% and 29% reduction, respectively) compared to cells transfected with non-targeting CT siRNA. Treatment with 10 nM E2 resulted in 2.2-fold increase in cell growth after 72 h in CT siRNA and 2.3-fold increase in β-catenin siRNA transfected cells. Based on the growth observed in non-stimulated CT siRNA-transfected cells, E2-treatment resulted in 1.6-fold increase in β-catenin siRNA transfected cells. The growth reduction after β-catenin knockdown in cells not treated with E2 suggests an E2/ERα independent effect of β-catenin on cell proliferation. It is well known that β-catenin is in the hub of cross-talk between multiple cellular pathways and has important roles in the regulation of cell growth induced by different stimuli [21]. For example, β-catenin can activate cell proliferation by multiple mechanisms including the canonical Wnt signaling pathway [20] or induction of cell cycle genes like cyclin D1 [21]. Activation of the Wnt pathway can be excluded for MCF-7 cells because this cell line does not express functional Wnt receptors. Cyclin D1 is not only a target gene of β-catenin, but also of ERα [22]. This applies also to other growth related genes which complicates the experimental analysis of their role in ERα/β-catenin crosstalk.

We know from our previous work that in MCF-7 cells ERα always has a certain basal activity, even in the absence of the ligand [8,9]. This may be due to ligand-independent ERα activation by compounds present in the culture medium. Active ERα stimulates MCF-7 cell proliferation. Therefore, we assume that the reduction of cell growth after β-catenin silencing in cells not treated with E2 may at least be partly due to reduced ERα protein. The partial inhibition of E2-induced cell growth by β-catenin knockdown shown in Figure 5 may therefore be explained by the reduction of ERα levels under these conditions. The only partial reduction of E2-induced cell growth obtained by β-catenin knockdown permits the conclusion that β-catenin is only one of the many regulators or modulators of ERα function.

The vast majority of newly diagnosed human breast cancers are ERα positive, although the number of positive cells may vary significantly. The expression level of ERα is the most important determinant for regulation of the transcriptional activity of the receptor. Any mechanisms resulting in changes of ERα levels result in altered expression of ERα target genes. Besides, a number of cellular signaling mechanisms contribute to the activity of ERα. Many genes that are positively regulated by ERα are related to cell proliferation. In this study we have demonstrated that β-catenin knockdown resulted in reduced ERα mRNA and protein expression, indicating β-catenin as a positive regulator of ERα expression levels. Furthermore, β-catenin knockdown resulted in reduced E2 stimulated gene expression and cell growth. The lacking direct physical interaction between β-catenin and ERα indicates that β-catenin is not a coactivator of ERα in breast cancer cells. The data presented suggest that β-catenin rather acts as a transcription factor regulating ERα expression. Because β-catenin is frequently overexpressed in human breast cancer, it may be speculated that this cross-talk between β-catenin and ERα is a determinant of E2-dependent breast cancer cell proliferation.

## 3. Experimental

### 3.1. Cell Lines and Materials

MCF-7 cells (from DSMZ, Braunschweig, Germany) and MELN cells (derived from MCF-7 cells by stable transfection with an ERE-controlled luciferase reporter plasmid) [23] were maintained routinely (5% CO_2_, 37 °C, 100% humidity) in phenol red-free high-glucose DMEM (Invitrogen, Karlsruhe, Germany) supplemented with 10% fetal calf serum (FCS, Biochrom, Berlin, Germany), 100 U/mL penicillin and 100 μg/mL streptomycin (Biochrom). BT-474 and T-47D (from ATCC/LGC, Wesel, Germany) were maintained in RPMI-1640 (PAA, Cölbe, Germany) supplemented with 10% FCS and 100 U/mL penicillin and 100 μg/mL streptomycin. Four days before experimental use cells were kept in medium with 10% dextran-coated charcoal-treated FCS (DCC-FCS, prepared as described [24]).

Mouse monoclonal antibodies used were against ERα, (Novocastra, Newcastle, UK), β-catenin, eIF4B, histone H3 (all from Cell Signaling Technologies, New England Biolabs, Frankfurt/Main, Germany), β-actin (Abcam, Cambridge, UK). CT4 non-targeting siRNA sequence was from Dharmacon (Boulder, CO, USA, Cat # D-001810-04-05), β-catenin targeting siRNA was from Santa Cruz Biotechnology (Heidelberg, Germany) and ERα siRNA from New England Biolabs. Oligofectamine was from Invitrogen (Karlsruhe, Germany), protein A agarose beads from Roche (Mannheim, Germany), and 17β-estradiol (E2) from Sigma (München, Germany). Protein concentrations of cell lysates were determined with the DC protein assay kit from Bio-Rad (München, Germany).

### 3.2. Subcellular Fractionation

5 × 10^6^ MCF-7 cells were plated in 10 cm dishes in DMEM containing 10% DCC-FCS and treated with 10 nM E2 for 20 or 30 min. Cells were then trypsinized and centrifuged at 500× g for 10 min at 4 °C. Pellets were washed in ice cold PBS (phosphate-buffered saline, Mg^2+^ and Ca^2+^ free). Thereafter, subcellular fractionation of the cells was performed using the Qproteome Cell Compartment kit (Qiagen, Hilden, Germany) following manufacturer's manual. Western blot analysis was performed as described [8,9], eFI4B and H3 were used as loading control for the cytoplasmic and nuclear fraction, respectively.

### 3.3. Cell Proliferation Study

For studying cell growth, 1 × 10^4^ MCF-7 cells were plated in DMEM containing 10% DCC-FCS in each well of a 96-well plate. After 18 h, cells were transfected with 80 nM siRNA targeting β-catenin using Oligofectamine (2 μL/well). For control, cells were transfected with 80 nM non-targeting siRNA. After 24 h of transfection, cells were starved in DMEM containing 2% DCC-FCS for another 24 h. Thereafter cells were stimulated with 10 nM E2 for 72 h. At the end of the incubation time, the cells were washed with 100 μL of PBS, fixed for 15 min with 100 μL of 4% paraformaldehyde in PBS and stained for 30 min with 100 μL of 1% crystal violet dye dissolved in 10% ethanol. Excess crystal violet dye was removed and plates were extensively washed with water to remove traces of unbound crystal violet dye. After air drying, the bound dye was dissolved in 100 μL of 10% acetic acid. Optical density was read at 595 nm using Multiscan MX plate reader.

### 3.4. Transient Transfection of Cells with siRNA

3 × 10^5^ cells/well were seeded in 6-well plates. After 18 h cells were transfected with either 80 nM siRNA targeting β-catenin, ERα or non-targeting siRNA using oligofectamine and following the manufacturer's manual. 24 h and 48 h after transfection, cells were stimulated with 10 nM E2 for another 16 h. Thereafter, either RNA isolation or cell lysis was performed.

### 3.5. RNA Isolation and Quantification

Total RNA was isolated using the RNeasy kit (Qiagen). 1 μg of total RNA from each sample was reverse transcribed to cDNA using the QuantiTect Reverse Transcription Kit (Qiagen). qRT-PCR was conducted using a PTC-200 Peltier Thermal Cycler (MJ Research, Miami, FL) and the DyNAmo Flash SYBR Green qPCR kit (New England Biolabs). All qRT-PCR reactions were performed in triplicate. Melting curve analysis was performed to ascertain amplification of one specific gene product. Relative mRNA expression was calculated using the comparative Ct method (2^-ΔΔCt^) and normalized to the house keeping gene HMBS. The primers used were:

β-catenin fwd: 5′-AAT ACC ATT CCA TTG TTTG TGC AG-3′
β-catenin rev: 5′AGC TCA ACT GAA AGC CGT TT-3′
ERα fwd: 5′-TTA CTG ACC AAC CTG GCA GA-3′
ERα rev: 5′-ATC ATG GAG GGT CAA ATC CA-3′
pS2 fwd: 5′-ATA CCA TCG ACG TCC CTC CA-3′
pS2 rev: 5′-AAG CGT GTC TGA GGT GTC CG-3′
GREB 1 purchased from Qiagen (cat # QT00080262)
HMBS fwd: 5′-CGC ATC TGG AGT TCA GGA GTA-3′
HMBS rev: 5′-CCA GGA TGA TGG CAC TGA-3′

### 3.6. Luciferase Assay

MELN cells were transfected with siRNA and, after 48 h, were stimulated with 10 nM E2 for 16 h. Thereafter, cells were washed with ice cold PBS (Mg^2+^ and Ca^2+^ free), and lysed (30 min, 4 °C) using 150 μL/well of Luciferase Cell Culture Lysis Reagent (Promega, Mannheim, Germany). After centrifugation (10,000× g, 15 min, 4 °C), the supernatant was collected and luciferase activity was analyzed using the Bright Glo firefly luciferase assay system (Promega) and a Biolumat LB 9505 from Berthold (Bad Wildbad, Germany). Protein estimation of each sample was performed for normalization.

### 3.7. Immunoprecipitation

Protein-A agarose beads (50 μL) were processed by washing first twice in PBS and then twice with lysis buffer [8]. Protein (cell lysate, 700 μg) together with primary antibody or non-immune IgG (negative control) (2 μg) was added to the washed beads (final volume was 750 μL) and left overnight for incubation at 4 °C on a rotating shaker. Next morning, tubes were centrifuged and supernatant was preserved. Thereafter, the beads were washed 4 times with lysis buffer and finally twice with PBS. Beads were then suspended in 30 μL of 2.5× SDS buffer, boiled at 95 °C for 5 min to separate the protein complex from beads. The beads were centrifuged for 10 min and the supernatant was loaded and resolved on 10% SDS-polyacrylamide gel followed by immunoblotting for ERα and β-catenin on the same membrane.

### 3.8. Statistical Analysis

For quantitative analysis of Western blots, signal intensities were determined with the ImageJ software (National Institutes of Health, Bethesda,MD, USA) and were normalized with β-actin used as loading control. Luciferase analysis was performed using Berthold LB 9505 C (version 4.08) software. For analysis of qRT-PCR, MJ opticon monitor analysis software (version 3.1) from Bio-Rad was used. For each set of data, mean ± SEM was calculated evaluating three independent experiments. Differences between groups were statistically evaluated using Student's *t*-test. A P value < 0.05 was considered significant.

## 4. Conclusions

Our paper demonstrates an interaction between β-catenin and ERα signaling. We show that β-catenin is a positive regulator of ERα mRNA and protein expression levels and of ERα activity.

## Supplementary Files

Supplementary File 1 PDF-Document (PDF, 92 KB)
