# Supplementary material for: β-Catenin Is a Positive Regulator of Estrogen Receptor-α Function in Breast Cancer Cells
Source: Cancers (Basel). 2011 Jul 22;3(3):2990–3001. doi: 10.3390/cancers3032990 (PMC3759182; doi:10.3390/cancers3032990)
Supplement: Supplementary File 1 — PDF-Document (PDF, 92 KB) [file cancers-03-02990-s001.pdf]

Cancers **2011**, *3*, 2990-3001; doi:10.3390/cancers3032990

OPEN ACCESS

cancers

ISSN 2072-6694
www.mdpi.com/journal/cancers

Supplementary file

β-Catenin Is a Positive Regulator of Estrogen Receptor-α Function in Breast Cancer Cells

Nibedita Gupta, Fee Schmitt, Sina Grebhardt and Doris Mayer *

Hormones and Signal Transduction Group, German Cancer Research Center (DKFZ), DKFZ-ZMBH Alliance, Im Neuenheimer Feld 581, 69120 Heidelberg, Germany; E-Mails: n.gupta@dkfz.de (N.G.); fee.schmitt@gmx.de (F.S.); s.grebhardt@dkfz.de (S.G.)

* Author to whom correspondence should be addressed; E-Mail: d.mayer@dkfz.de; Tel.: +49-6221-423238; Fax: +49-6221-423237.

Received: 9 June 2011; in revised form: 18 July 2011 / Accepted: 19 July 2011 / Published: 22 July 2011

Figure S1. ERα and β-catenin do not co-immunopreciptitate from lysates of T-47D cells. T-47D cells were grown in DMEM containing 10 % FCS. ERα was immunoprecipitated (IP) from cell lysates. The immunoprecipitate (IP) contains ERα but β-catenin was not co-precipitated. Neg Con, negative control containing non-immune IgG. Input: cell lysate.

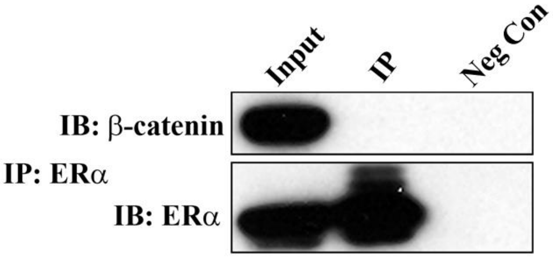

© 2011 by the authors; licensee MDPI, Basel, Switzerland. This article is an open-access article distributed under the terms and conditions of the Creative Commons Attribution license (http://creativecommons.org/licenses/by/3.0/).
